# Folding Very Short Peptides Using Molecular Dynamics 

**DOI:** 10.1371/journal.pcbi.0020027

**Published:** 2006-04-14

**Authors:** Bosco K Ho, Ken A Dill

**Affiliations:** Department of Pharmaceutical Chemistry, University of California San Francisco, San Francisco, California, United States of America; Weill Medical College of Cornell University, United States of America

## Abstract

Peptides often have conformational preferences. We simulated 133 peptide 8-mer fragments from six different proteins, sampled by replica-exchange molecular dynamics using Amber7 with a GB/SA (generalized-Born/solvent-accessible electrostatic approximation to water) implicit solvent. We found that 85 of the peptides have no preferred structure, while 48 of them converge to a preferred structure. In 85% of the converged cases (41 peptides), the structures found by the simulations bear some resemblance to their native structures, based on a coarse-grained backbone description. In particular, all seven of the β hairpins in the native structures contain a fragment in the turn that is highly structured. In the eight cases where the bioinformatics-based I-sites library picks out native-like structures, the present simulations are largely in agreement. Such physics-based modeling may be useful for identifying early nuclei in folding kinetics and for assisting in protein-structure prediction methods that utilize the assembly of peptide fragments.

## Synopsis

To carry out specific biochemical reactions, proteins must adopt precise three-dimensional conformations. During the folding of a protein, the protein picks out the right conformation out of billions of other conformations. It is not yet possible to do this computationally. Picking out the native conformation using physics-based atomically detailed models, sampled by molecular dynamics, is presently beyond the reach of computer methods. How can we speed up computational protein-structure prediction? One idea is that proteins start folding at specific parts of a chain that kink up early in the folding process. If we can identify these kinks, we should be able to speed up protein-structure prediction. Previous studies have identified likely kinks through bioinformatic analysis of existing protein structures. The goal of the authors here is to identify these putative folding initiation sites with a physical model instead. In this study, Ho and Dill show that, by chopping a protein chain into peptide pieces, then simulating the pieces in molecular dynamics, they can identify those peptide fragments that have conformational biases. These peptides identify the kinks in the protein chain.

## Introduction

Peptide fragments of proteins often have intrinsic propensities for the formation of their native conformations. For example, NMR experiments [1] show that long peptide fragments have native-like conformations [2–7]. Some short peptides in solution have also been shown to adopt their native secondary structures: α helices [8,9] and β hairpins [10–14].

As a consequence, peptide conformational propensities that are taken from the protein databank (PDB) [1–17] are now widely used in protein-structure prediction algorithms. A popular set of peptide fragment conformations is the I-sites library of David Baker and his co-workers [18,19]. Extensive libraries of peptide fragments have now been compiled [20–22] and have become essential elements in protein-prediction methods [23]. From the recent CASP protein–structure prediction competition, it was noted that most of the successful de novo methods use a fragment-based approach [23,24]. Typically, a candidate protein native structure is spliced together from fragments that are extracted from a database of conformations, and then treated to conformational scoring and optimization.

Can physical models capture these conformational propensities of peptides? There is good evidence that they can. First, simple physical models can reproduce the structural biases of certain peptide fragments [25–28]. To date, however, such studies have largely focused on selected peptides that are expected to fold. Our interest here is to know whether physical models can also discriminate peptides that fold from peptides that do not. Second, in molecular dynamics simulations of small peptides, the ensemble of conformers divides into well-defined clusters. This has been found for a penta–β peptide in explicit water [29,30], and for a small α-helical peptide [31]. Third, molecular dynamic simulations of small peptides reproduce the α-helical propensities of certain fragments from the I-sites sequence-structure library [32]. Many models of protein folding kinetics assume that peptide fragments of the chain that have preferred conformations are responsible for nucleating the folding process [33–35].

Here, we study 133 peptide 8-mer fragments from six different proteins of different folds, using replica-exchange molecular dynamics sampling [36] in Amber7, with the parm96 parameters and the GB/SA (generalized-Born/solvent-accessible electrostatic approximation to water) implicit solvent model of Tsui and Case [37]. We chose this force field as it is the only implicit-solvation model that can adequately reproduce the native state of the β hairpin of protein G [38].

We are interested in whether this physical model can identify native-like secondary structures in peptide fragments. If so, it indicates the importance of local interactions in those cases. Our study involves complete coverage of those proteins. For each protein, we systematically generate a series of 8-mer peptide fragments with overlapping sequences from the original protein sequence. Neighboring fragments have a five-residue overlap (and three-residue gap). We chose 8-mers because this length appears adequate to identify elements of structure in PDB studies [19] and because much longer fragments become too expensive for computer simulations. We simulate each peptide using 16 replicas for 5 ns/replica, and keep only the last 1 ns.

In each case, we determine whether the peptide has converged to its native conformation in the folded protein. We consider two measures of convergence. First, we monitor the RMSD between the simulated conformations and the experimental PDB structure of that peptide. However, for prediction purposes—determining whether a peptide has a converged structure in the absence of knowledge of its native structure—we develop another measure based on the *backbone mesostring,* which is a coarse-grained description of the backbone conformational ensemble.

A mesostring is a one-dimensional list of the mesostates of each residue in a peptide. A mesostate refers to a discrete region of the φ–ψ angles of the backbone of a residue. Mesostate [a] corresponds to a helical conformation, including the α helix, 3_10_ helix, or π helix. Mesostate [b] corresponds to an extended β-strand conformation. Mesostate [l] corresponds to a left-handed helical conformation.

We use the mesostrings to cluster conformations in our simulations. Based on the three mesostates described above, an 8-mer has 3^8^ = 6,561 possible mesostrings. When each simulation is completed, each 8-mer peptide will have different populations for the 6,561 mesostrings, hence different free energies. The mesostring that represents the highest population (the lowest free energy) is called the *ground mesostring.* We use the properties of the ground mesostring to determine structural bias in a peptide. The ground mesostrings are classified in terms of either a reverse-turn or a helical-turn conformation (see Figure 1). We define a helical-turn as a mesostring that contains at least four [a] mesostates in a row, and a reverse-turn as a mesostring that contains either the [bab] or [baab] motifs.

**Figure 1:**
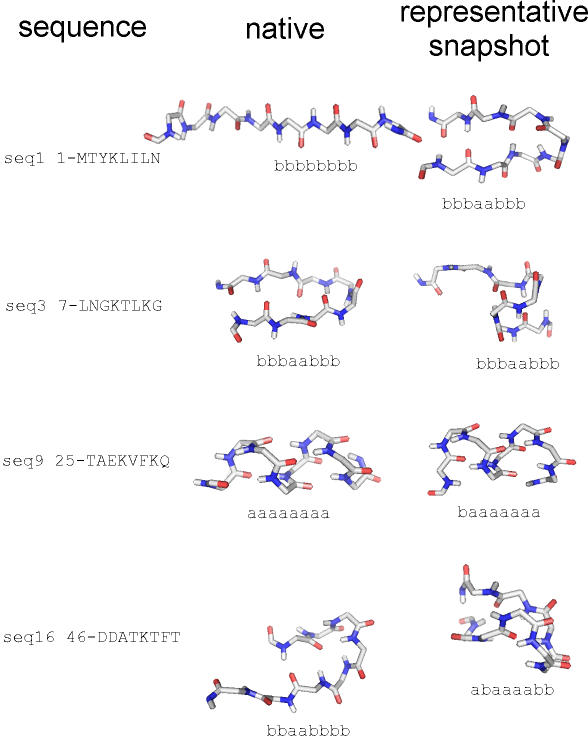
Representative Snapshots of Various Peptides from Protein G The representative snapshot is the snapshot in the ground mesostring with the lowest energy. The ground mesostring of seq1 and seq3 are classed as reverse-turns, and the ground mesostring of seq9 and seq16 are classed as helical-turns.

How do we know when a simulation has converged? We calculate the backbone entropy using the Boltzmann formula S = −k Σ_i_ p_i_ ln p_i_, where p_i_ is the probability that the peptide is in mesostring i. The backbone entropy is calculated over a certain window in a trajectory, where the sum is made over only the mesostrings that are observed in the window. The backbone entropy S is useful for two purposes. First, it measures for a given peptide the sharpness of the distribution of probabilities of the mesostrings. The more peaked the distribution is, and thus the more favored a mesostring is, the lower is the backbone entropy. In this way, the backbone entropy indicates whether any one conformation is substantially favored over the others, for the given peptide. Second, the backbone entropy should converge at equilibrium, approaching an asymptotic value with time in the simulation. Even if a new mesostring emerges late within the sampling (as is often the case), it only changes the backbone entropy if it has a significant population. We use the convergence of the backbone entropy to indicate the convergence of the simulation.

We study peptide fragments extracted from a series of well-characterized proteins: protein G, protein L, protein A, and α-spectrin, and chymotrypsin inhibitor. For each peptide, we simulate the ensemble of states at equilibrium. We find that some of these peptides exhibit strong structural biases. We analyze the relationship of those structural biases to the topology of the native structure.

## Results/Discussion

### Structural Bias in the Peptide Conformation Ensemble

Do peptides have native-like conformations? Figure 2 shows the simulated free-energy profiles of RMSD for the peptides of protein G. We call the region of RMSD < 2 Å native-like. We find that some fragments spend a significant amount of time near their native structures (seq3, seq9, and seq10). Some peptides have a broad conformational distribution (seq14), while others have a narrow distribution (seq16). Narrow distributions indicate structural bias in the peptide. To investigate this structural bias further, we list in Table 1 the lowest free-energy mesostrings of several protein G peptides. We show in Figure 1, a representative conformation of the ground mesostrings of these peptides.

**Figure 2:**
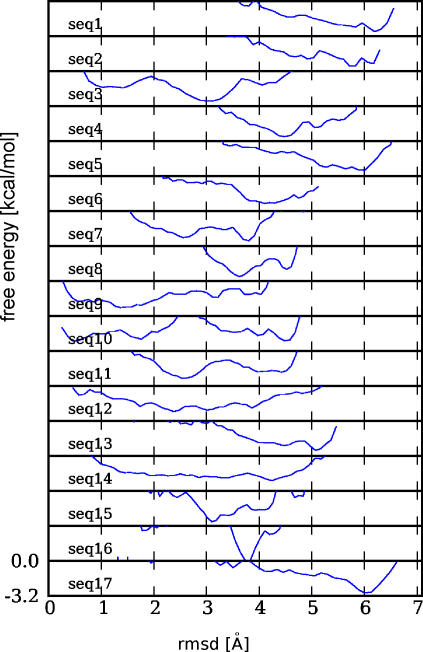
Free-Energy Profile of RMSD of Peptides from Protein G

**Table 1:**
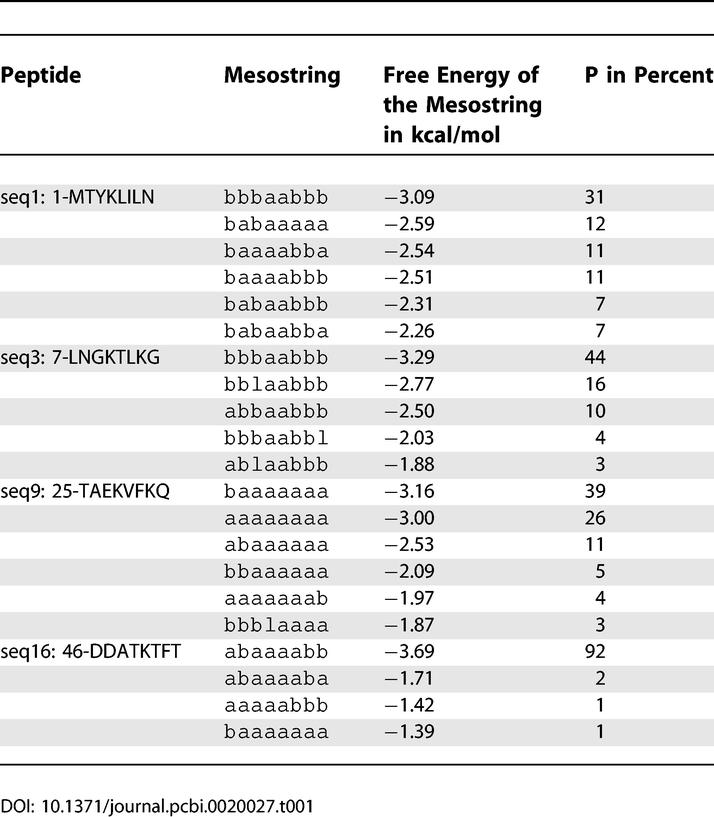
Mesostrings of Various Peptides from Protein G

Figure 3 shows the variation in backbone entropy for the peptides of protein G. To calculate the variation in Figure 3, we deliberately chose a smaller window (0.2 ns) than the window used for the analysis (1 ns in Tables 1–4) to emphasize the fluctuations. In most of the peptides, the backbone entropy equilibrates almost immediately, with the exception of seq16, which decreases to a near zero value at about 3.5 ns. Consequently, we carry out the main analysis of the structural bias over the last 1 ns of our 5-ns trajectories. The backbone entropy specifically measures the conformation freedom in the backbone. Backbone entropy is a useful measure only when the free-energy basins in phase space are dominated by the local conformation of the backbone, and not by nonlocal interactions. As these peptides are short, nonlocal interactions should be minimal, and the backbone entropy should be the dominant entropy.

**Figure 3:**
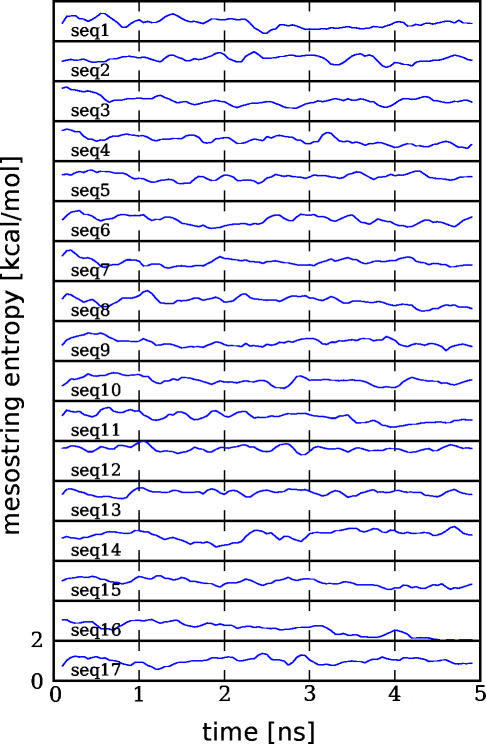
Variation of the Information Entropy of Mesostrings in the 17 Peptides from Protein G The entropy at each point is calculated over a 0.2-ns window. The backbone entropy holds fairly steady after 1 ns.

We define the existence of structural bias in a peptide in terms of two properties of the ground mesostring. First, we use the probability P_1_ in observing the ground mesostring, which is derived from the relative free energies. Second, we use the free-energy gap ΔF between the ground mesostring and the next mesostring to measure the relative probability of the ground mesostring from all the other mesostrings. Specifically, we consider a peptide to have structural bias if P_1_ > 45% and ΔF > 0.6 kcal/mol. Of the 133 peptides we studied, we found that 48 peptides have structural bias (bold in Tables 2–4). We refer to such peptides as *structured peptides*.

**Table 2:**
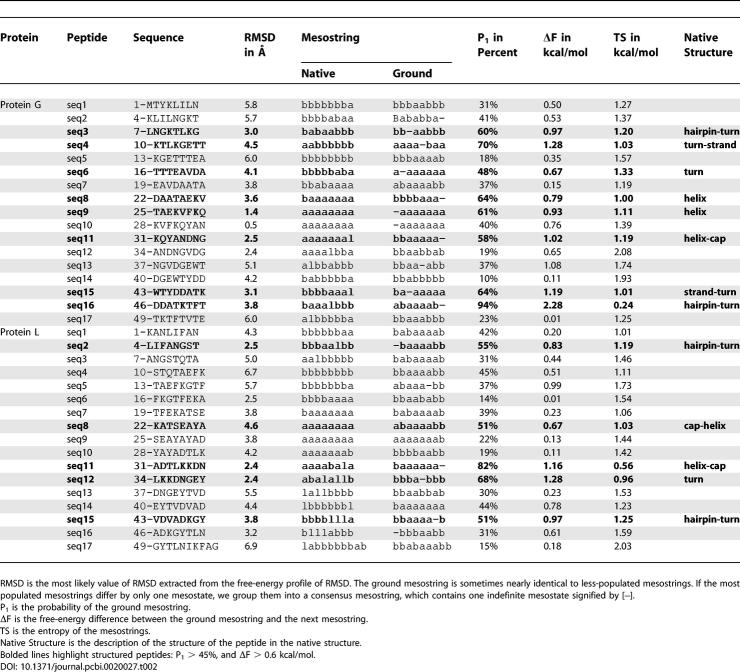
Ground Mesostrings of Protein G and Protein L

**Table 3:**
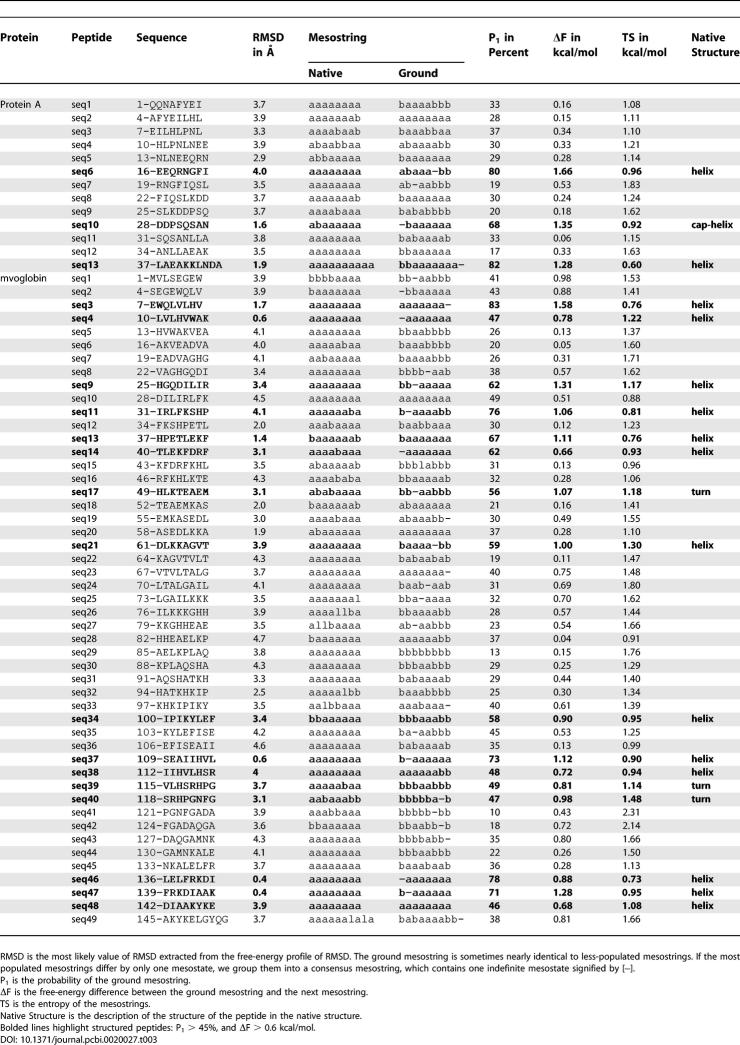
Ground Mesostrings of α-Helical Proteins

### Comparison of the Peptide Conformations with Native Structures

What parts of the native structure are picked out by the structured peptides? In Table 5, we list the ground mesostrings of the peptides in simulation. We highlight (in bold) the sequences that are structured and compare these structured peptides to the native secondary structures. The structured peptides adopt either a helical-turn or reverse-turn. Figure 4 shows the location of the structured peptides within the native fold topology. Below we describe the relationship between the structured peptides, the native structure, and experimental studies of the folding of these proteins.

**Table 4:**
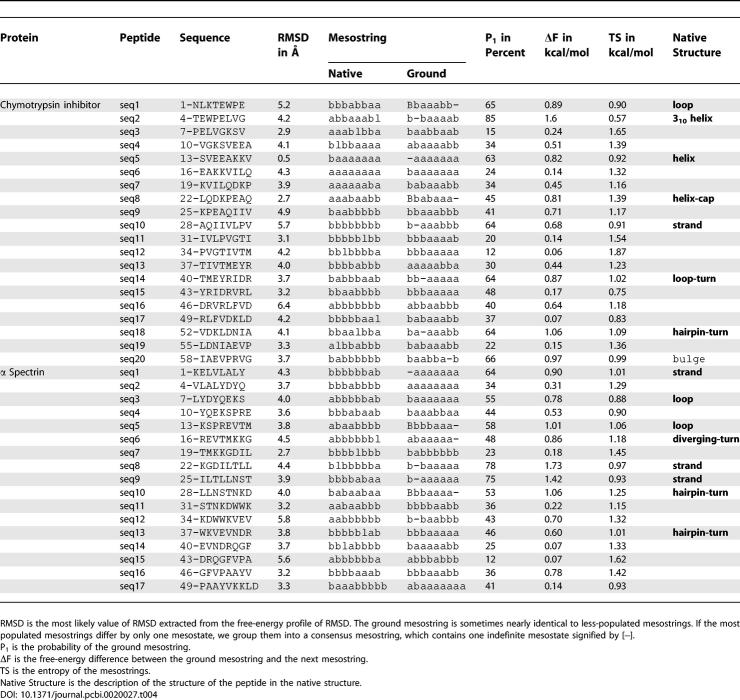
Ground Mesostrings of β-Sheet Proteins

**Table 5:**
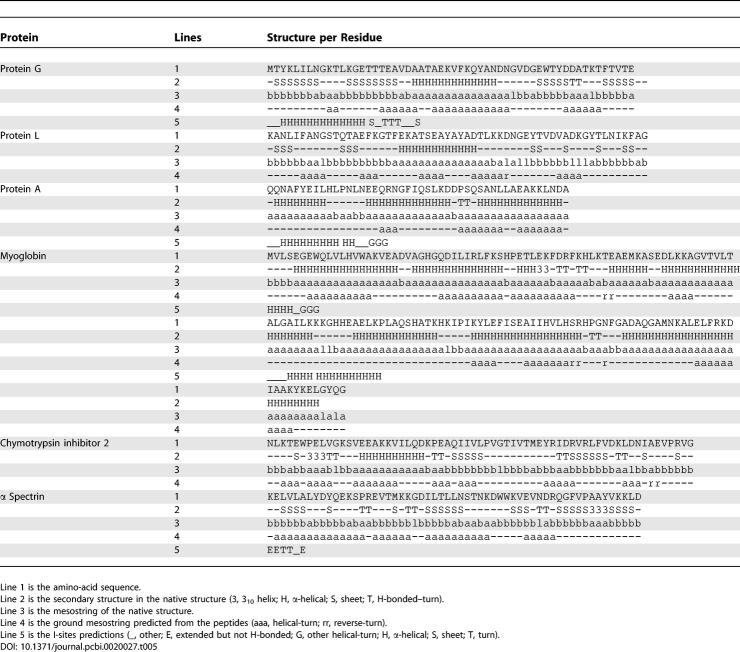
Comparison of the Structural Bias with the Native Structure

**Figure 4:**
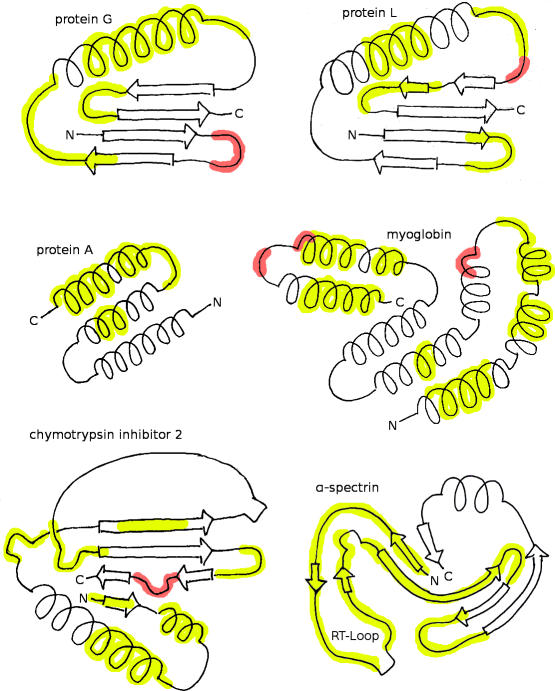
Distribution of Kinks in the Topology of Protein Structures α helices and 3_10_ helices are drawn as coils, hydrogen-bonded β-turns are drawn as a notch, and β strands are drawn as arrows. Yellow indicates helical-turn [-aaa-] and red indicates reverse-turns [baab]. 11 of the 14 α helices contain a helical-turn. The turns of all seven β hairpins contain a peptide fragment that is structured.

In the protein G fragments, we find eight structured peptides that adopt a stable helical-turn conformation (Table 2). Three of these helical-turns pick out the lone α helix in the native structure, another helical-turn picks out the turn between the helix and N-terminal β hairpin, and the remaining two helical-turns pick out the turn in the C-terminal β hairpin. Another two structured peptides with overlapping sequences adopt a stable reverse-turn conformation, which both pick out the same N-terminal hairpin-turn in the native structure. The isolated C-terminal β hairpin has been found experimentally to be stable [10], where this stability is reflected in the structural bias found in the peptide fragments of the hairpin-turn. The structured peptides provide an explanation for an ingenious study of secondary structure in protein G [39]. In that experiment, Minor and Kim replaced the α-helix sequence with a sequence based on the C-terminal hairpin. The mutant was able to fold into the same topology, showing that there is a helical propensity in the C-terminal hairpin. In the peptide studies, we find helical-turns in both the α helix and the turn of the C-terminal hairpin, which demonstrates the interchangeability of these two sequences in our simulations.

In the protein L fragments, we find four structured peptides that adopt a stable helical-turn conformation (Table 2). Two of the helical-turns pick out the α helix, while the other two helical-turns pick out the two hairpin-turns in the native structure. Another structured peptide adopts a reverse-turn conformation, which picks out the C-cap of the α helix.

In the fragments of the B domain of protein A, we found three structured peptides that adopt a stable helical-turn conformation (Table 3). These helical-turns pick out helix II and helix III, and the turn between these helices. The stability of these pieces is consistent with experimental studies of protein A fragments, which show that helix II and helix III form a stable intermediate [40].

In the myoglobin fragments, 13 structured peptides adopt a stable helical-turn conformation (Table 4). These helical-turns pick out six of the eight α helices in the native structure—with particularly long helical-turns in helices A, G, and H. Another three structured peptides adopt a stable reverse-turn conformation. Two of the reverse-turns pick out the same turn between helices G–H. The large amount of structural bias found in the fragments of helices G and H is consistent with experimental studies, which show that helices G and H form a stable intermediate [41]. Experimentally, helix F has the weakest helical propensity, and correspondingly we do not find any structured peptides in fragments of helix F.

In the chymotrypsin inhibitor fragments, we found eight structured peptides that adopt a stable helical-turn conformation (Table 4). One helical-turn picks out the 3_10_ helix in the native structure, two helical-turns pick out the α helix, one helical-turn picks out a diverging turn, and one helical-turn picks out the turn in the β hairpin. Two helical-turns erroneously pick out β strands. We also found a structured peptide that adopts a reverse-turn conformation. This reverse-turn picks out the bulge in a β strand. Experimental studies find that only the α helix is stable [42].

In the α*-*spectrin fragments, there are eight structured peptides that adopt stable helical-turn conformations. Two of the helical-turns erroneously pick out the RT loop. The conformation of the RT loop is somewhat indeterminate as both experimental and simulation studies (unpublished data) show that the RT loop is unstable. Another helical-turn overlaps with a diverging β-turn in the native structure. Three helical-turns erroneously pick out a β strand. The other two helical-turns pick out the turns of the two β hairpins. Experimental studies find that only a fragment of the last β hairpins has structure in solution [43].

Overall, of the 41 structured peptides that adopt a stable helical-turn conformation, 21 pick out α helices, three pick out 3_10_ helices, and two overlap with diverging turns. Because helical motifs can be considered a continuum from diverging β-turns, to 3_10_ helices, to α helices [44,45], we conclude that 26 of the helical-turns pick out helical motifs in the native structures. Another seven helical-turns pick out β-hairpin–turns, and one helical-turn is found in a helix hairpin-turn. Five helical-turns erroneously pick out β strands and two other helical-turns erroneously pick out the RT loop.

We find six structured peptides that adopt a reverse-turn conformation: one is found at a hairpin turn, two are found at strand–helix turns, three are found at helix–helix turns, and one is found at a β-strand bulge.

There is some debate [46] over whether β hairpins fold via the turn [47] or through hydrophobic clustering [48]. The results here suggest that structural bias at the turn is very important. We find that all seven β hairpins in the six proteins contain a fragment in the turn that results in a structured peptide. If we interpret the structural bias in the peptide as a kink in the full chain, then the formation of structure can be regarded as contacts coalescing around a kinky chain. In terms of the β hairpin, this does not necessarily mean that the turn forms first but that a kink favors the formation of nearby contacts.

In summary, of the 48 structured peptides found in the simulations, only five differ significantly from the native structure. Given that there are 436 residues in our six proteins, there is, on average, a kink (secondary structural indicator) approximately every nine residues along the chain.

### Comparison with the I-Sites Library

Do the structural biases that are found in our simulations correlate with those in the PDB? We focus on the I-sites server (http://www.bioinfo.rpi.edu/~bystrc/hmmstr/server.php), a fragment database that predicts the structures of short protein sequences [19]. In that database, predictions that have a high confidence score (>0.8) are found to predict a structure that is <1.4 Å from the native structure with a 74% probability. I-sites make eight such high-confidence predictions over four of the proteins in our dataset. Table 5 shows those successes of I-sites. Our structured peptides overlap with the I-sites predictions in six of the eight I-sites predictions. This suggests that the I-sites sequence-structure correlations are at least partly encoded in the local structural biases found in the structured peptides.

### Conclusion

In this study, we have applied replica-exchange molecular dynamics, using the parm96 force field with a GB/SA solvent model, to the simulation of 133 peptide 8-mer fragments, extracted from six proteins with five different folds. We found that 48 of these peptides are strongly structured. The remaining 85 peptides have no preferred structure. Of the 48 that are structured, 41 of them fold into approximately their native conformations. In seven instances, the simulated structures are significantly inconsistent with their native structures.

Why are only 35% of the peptides structured? The reason is that by using very short peptides, we have eliminated most of the nonlocal interactions—hydrophobic clustering, cooperative helical hydrogen bonds. We thus attribute any structural bias to sidechain interactions, which will depend on specific sequence motifs.

As with all molecular dynamics simulations, the results will depend somewhat on the choice of force field. One limitation, for example, is that none of the current force fields model the backbone very well, especially in glycine. Neither can current force fields model the left-handed α-helical conformation accurately, resulting in the paucity of ground mesostrings containing the [l] mesostate. Better force fields may improve our predictions. As we simulated only short peptides, we have eliminated various cooperative nonlocal interactions—interactions that are particularly sensitive to specific details of the force field.

The I-sites library taken from PDB peptide preferences makes eight high-confidence predictions in four of the six proteins. In those instances, our simulations are largely consistent with theirs, indicating that the intrinsic physical preferences contribute to the PDB structures. However, the present simulations are also more informative, giving 48 structures (with 85% reliability) among the 133 peptides we tested, in contrast to the eight (having 74% reliability) found by I-sites.

Current structure-prediction systems rely on a pragmatic mix of bio-informatics and physical modeling [23,24]. A key component of these systems is the use of fragment libraries to identify folding initiation sites. Here we have identified the physical origin of the sequence–structure relations identified in the fragment libraries—local structural bias in short peptide sequences. The calculations are not exorbitant, as each peptide takes ~160 CPU node hours, and, in many cases, our results go beyond the fragment libraries. By replacing fragment libraries with peptide simulations to identify folding initiation sites, we move closer to the goal of predicting protein structures using only physical models.

## Materials and Methods

### Replica-exchange simulations of the peptides.

Replica-exchange simulations were conducted using a PERL wrapper (http://www.dillgroup.ucsf.edu/~jchodera/code/rex) around the SANDER molecular dynamics program for the Amber7 molecular-modeling package [49]. We used 16 replicas exponentially spaced between 270K and 690K, achieving an exchange–acceptance probability of approximately 50%. Exchanges were attempted every 1 ps, with constant-energy dynamics conducted between exchanges. After each exchange attempt, the velocities were redrawn from the appropriate Maxwell-Boltzmann distribution to ensure proper thermostating. A 2-fs time step was used, and bonds to hydrogens were constrained with SHAKE [50]. Configurations were stored every 1 ps for analysis. Simulations were run for 5 ns per replica and the first 4 ns were used for equilibration. The peptides were capped with ACE and NME blocking groups, and initialized in the extended state. Systems were set up using the LEAP program. Peptide parameters were taken from the Amber Parm96 force field, and the GB/SA model of Tsui and Case was used [37], along with a surface area penalty term of 5 cal · mol^−1^ · Å^−2^.

### Calculating thermodynamic observables.

We use replica exchange [36] to simulate the equilibrium ensemble. It samples k parallel replicas, each of which is at a different temperature. Hence, to extract thermodynamic observables for a given temperature, say T = 300K, we must reweigh the configurations taken from the k different temperatures β^k^ in order to combine them into a representative ensemble. We do this reweighing of the replicas with an implementation [51] of the Weighted Histogram Analysis Method [52].

We first calculate the dimensionless free-energy f^k^ for each replica k. Starting with a crude estimate of f^k^, we calculate Ω^k^_E_—the weight of states with energy E in replica k:

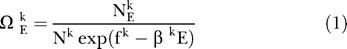
where N^k^_E_ is the number of snapshots in replica k with energy E. From the distribution of Ω^k^_E_, we calculate a new estimate of f^k^ by

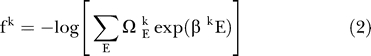



We iterate the above two steps until f^k^ converges. Then we use these dimensionless free energies f^k^ to reweigh the relative free-energy profile F of observable x to the target temperature β_tar_:

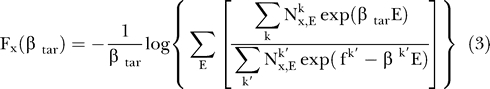



After using the Weighted Histogram Analysis Method to calculate the relative free energies F_i_ of a mesostring i, we calculate the probabilities P_i_ by

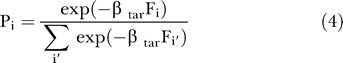



When we merge similar mesostrings into a consensus mesostring, we calculate the free-energy difference to another mesostring j by

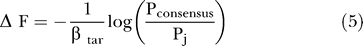



### Defining the backbone mesostates.

A key part of our analysis is the discretizing of the backbone degrees of freedom. This is based on the original analysis of the protein backbone [53]. In that analysis, Ramachandran and coworkers showed that the stereochemistry of the protein backbone breaks up the backbone ϕ–ψ angles into three distinct regions, each separated by significant energy barriers. We can thus describe the conformation of a peptide as a string of discrete mesostates—we call this the mesostring. A given mesostring is separated in energy from other mesostrings. Each mesostring corresponds to a low-energy basin in the conformation space of the peptide backbone. It is then straightforward to extract the local structure from the lowest free-energy basin. This partitioning in terms of discrete regions in the backbone angles has been observed in a molecular dynamics simulation of an α-helical peptide [31].

The original analysis of the backbone identified three distinct regions in the ϕ–ψ angles [53]. Recent studies of the protein database found that these three regions can be further divided up into five clusters of density [54,55]. Some of the barriers between these five regions are small, which leaves three regions separated by large barriers. However we cannot use the database analysis to define the boundaries of the backbone mesostates because current force fields cannot replicate the database distribution of ϕ–ψ angles. We must define the boundaries the backbone mesostates in terms of the force field in our molecular dynamics: we ran replica-exchange simulations of the alanine dipeptide and the glycine dipeptide for 10 ns and calculated the free-energy profile of the ϕ–ψ angles in bins of 5°. Based on the resultant free-energy profile, we break up the Ramachandran plot in terms of the following mesostates:


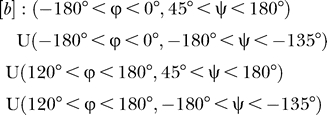



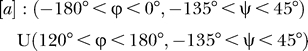



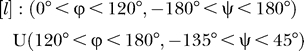


And for glycine:


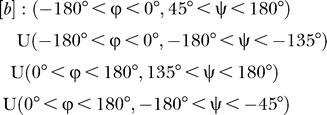



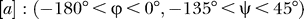



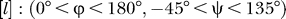


## Abbreviations

GB/SA: generalized-Born solvent-accessible electrostatic approximation to water
PDB: protein databank
RMSD: root-mean-square deviation
