# Correction: Folding Very Short Peptides Using Molecular Dynamics

**DOI:** 10.1371/journal.pcbi.0020060

**Published:** 2006-05-26

**Authors:** Bosco K Ho, Ken A Dill

In *PLoS Computational Biology,* volume 2, issue 4: DOI: 10.1371/journal.pcbi.0020027

The abbreviation GB/SA had an incorrect definition in the Abstract, Introduction, and Abbreviations list. The correct definition of GB/SA is generalized-Born/surface area.

Table 4 has several rows that did not appear in bold font in the published article, and Table 5 had four rows with incorrect spacing in the published article. Both tables appear correctly below.

**Table 4:**
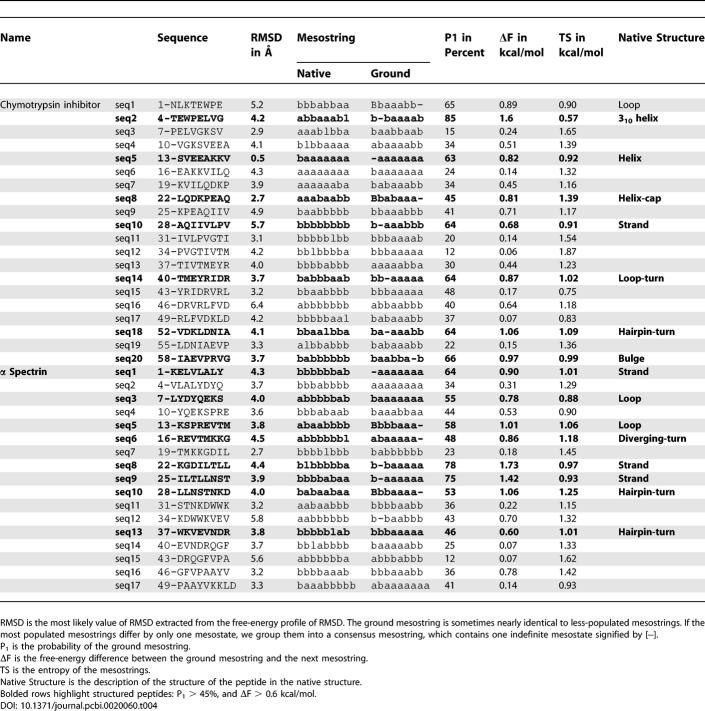
Ground Mesostrings of β-Sheet Proteins

**Table 5:**
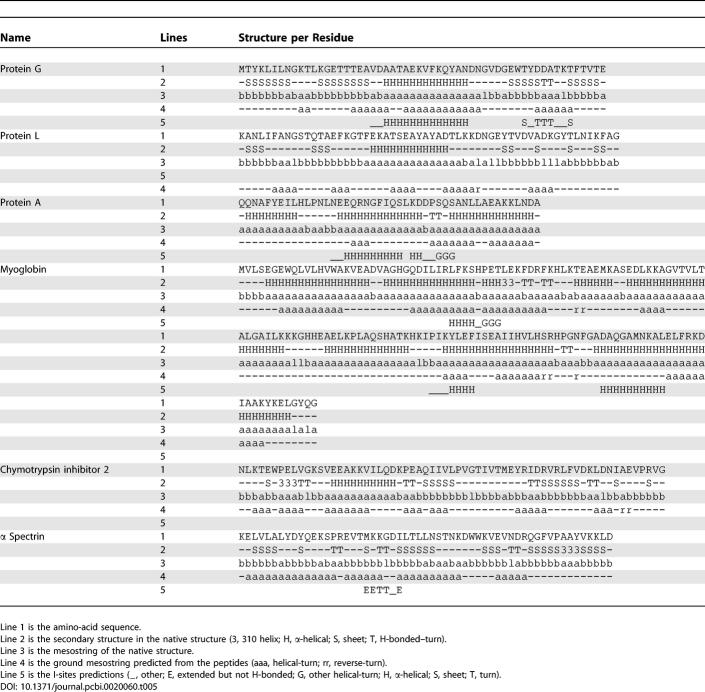
Comparison of the Structural Bias with the Native Structure

